# Insights into the Activity and Substrate Binding of *Xylella fastidiosa* Polygalacturonase by Modification of a Unique QMK Amino Acid Motif Using Protein Chimeras

**DOI:** 10.1371/journal.pone.0142694

**Published:** 2015-11-16

**Authors:** Jeremy G. Warren, James E. Lincoln, Bruce C. Kirkpatrick

**Affiliations:** Department of Plant Pathology, University of California Davis, Davis, California, United States of America; University of the West of England, UNITED KINGDOM

## Abstract

Polygalacturonases (EC 3.2.1.15) catalyze the random hydrolysis of 1, 4-alpha-D-galactosiduronic linkages in pectate and other galacturonans. *Xylella fastidiosa* possesses a single polygalacturonase gene, *pglA* (PD1485), and *X*. *fastidiosa* mutants deficient in the production of polygalacturonase are non-pathogenic and show a compromised ability to systemically infect grapevines. These results suggested that grapevines expressing sufficient amounts of an inhibitor of *X*. *fastidiosa* polygalacturonase might be protected from disease. Previous work in our laboratory and others have tried without success to produce soluble active *X*. *fastidiosa* polygalacturonase for use in inhibition assays. In this study, we created two enzymatically active *X*. *fastidiosa / A*. *vitis* polygalacturonase chimeras, AX1A and AX2A to explore the functionality of *X*. *fastidiosa* polygalacturonase *in vitro*. The AX1A chimera was constructed to specifically test if recombinant chimeric protein, produced in *Escherichia coli*, is soluble and if the *X*. *fastidiosa* polygalacturonase catalytic amino acids are able to hydrolyze polygalacturonic acid. The AX2A chimera was constructed to evaluate the ability of a unique QMK motif of *X*. *fastidiosa* polygalacturonase, most polygalacturonases have a R(I/L)K motif, to bind to and allow the hydrolysis of polygalacturonic acid. Furthermore, the AX2A chimera was also used to explore what effect modification of the QMK motif of *X*. *fastidiosa* polygalacturonase to a conserved RIK motif has on enzymatic activity. These experiments showed that both the AX1A and AX2A polygalacturonase chimeras were soluble and able to hydrolyze the polygalacturonic acid substrate. Additionally, the modification of the QMK motif to the conserved RIK motif eliminated hydrolytic activity, suggesting that the QMK motif is important for the activity of *X*. *fastidiosa* polygalacturonase. This result suggests *X*. *fastidiosa* polygalacturonase may preferentially hydrolyze a different pectic substrate or, alternatively, it has a different mechanism of substrate binding than other polygalacturonases characterized to date.

## Introduction

Pierce’s disease (PD) of grapevine is caused by the gram negative plant pathogenic bacterium *Xylella fastidiosa* [1–3]. To induce disease, *X*. *fastidiosa* must be able to spread from one xylem element to another [4]. *X*. *fastidiosa* accomplishes this by enzymatically degrading the xylem pit membranes that separate adjacent elements; this degradation is primarily accomplished by an endopolygalacturonase that is encoded by a single gene in the bacterial genome [5–9]. Previous research showed that if the gene encoding *X*. *fastidiosa* polygalacturonase was disrupted, the resulting polygalacturonase deficient mutant was non-pathogenic in grapevines [5]. Additionally, work by Aguero et al. (2005) shows that a polygalacturonase inhibiting protein (PGIP) from pear is able to reduce PD severity when expressed in transgenic grapevines [10]. These results suggest that if grapevines expressed sufficient amounts of an inhibitor of *X*. *fastidiosa* polygalacturonase, then *X*. *fastidiosa* cells could not degrade the pit membranes separating xylem elements. This would prevent *X*. *fastidiosa* from systemically colonizing the plant and prevent PD symptom development.

Unfortunately, producing enough active soluble *X*. *fastidiosa* polygalacturonase has been a major impediment for performing polygalacturonase inhibition assays. Previous efforts in our lab and others to produce soluble active *X*. *fastidiosa* polygalacturonase have suffered from its insolubility in heterologous expression systems. In all recombinant protein expression systems tried to date, *X*. *fastidiosa* polygalacturonase is produced in an insoluble and inactive form. Fast protein liquid chromatography (FPLC) was utilized to attempt to solubilize and purify insoluble recombinant *X*. *fastidiosa* polygalacturonase produced in *Escherichia coli*, however, this method did not produce an enzymatically active form of *X*. *fastidiosa* polygalacturonase.

Another issue complicating the ability to produce enzymatically active *X*. *fastidiosa* polygalacturonase *in vitro* is the fact that *X*. *fastidiosa* polygalacturonase appears to have a unique a distinct substrate binding amino acid motif compared to other characterized active endopolygalacturonase enzymes. [6, 11–14]. Whereas *X*. *fastidiosa* polygalacturonase possesses all the conserved catalytic amino acids implicated in the hydrolysis of 1, 4-alpha-D-galactosiduronic linkages, it has very different amino acids in the substrate binding domains [6]. Specifically, the R/ (I/L/V)/K motif that is possessed by all other known active endopolygalacturonases, is QMK (380–382) in the *X*. *fastidiosa* polygalacturonase [11]. It was shown in previous studies with *Aspergillus niger* polygalacturonase that mutation of the R residue in the RIK (256–258) motif to an A results in only 14% polygalacturonase activity compared to the wild type polygalacturonase. More importantly, changing R256 to Q resulted in only 6.5% residual polygalacturonase activity [15, 16]. Thus, it is possible that the presence of the Q residue in the *X*. *fastidiosa* polygalacturonase could result in substantially lower activity compared to other characterized polygalacturonases, or alternatively it might be reasonably active but has a different substrate specificity than most other polygalacturonases. The experiments conducted in this study focused on two questions: first, can we produce active, soluble *X*. *fastidiosa* polygalacturonase *in vitro* and second, do the altered amino acids in the substrate binding motif of the *X*. *fastidiosa* polygalacturonase result in reduced enzyme activity, or different substrate specificity. We decided to address both of these questions through the creation of a protein chimera in which the catalytic and substrate binding domains of *X*. *fastidiosa* polygalacturonase were exchanged with those in an active polygalacturonase enzyme from a different prokaryotic plant pathogen. Previous research has shown that protein chimeras can be useful for studying enzymatic activity and substrate binding motifs [17, 18]. *Agrobacterium vitis* polygalacturonase is the same size as *X*. *fastidiosa* polygalacturonase, and is important in the virulence of *A*. *vitis* to grapevines [19, 20]. Relevant to this study, a soluble active form of *A*. *vitis* polygalacturonase can be readily produced in recombinant *E*. *coli* expression systems [20, 21].

The N-terminal and C-terminal regions of both *A*. *vitis* and *X*. *fastidiosa* polygalacturonase contain the greatest sequence variation while the middle (amino acids ~ 290–390), which contains the active site and substrate binding amino acids, is highly conserved between the two. [6, 20]. This suggests that the variable regions might be responsible for the inability to produce *X*. *fastidiosa* polygalacturonase in an active and soluble form in *in vitro* expression systems. Thus, a chimeric protein containing the variable N terminal and C terminal regions from the soluble and active *A*. *vitis* polygalacturonase, combined with the catalytic and substrate binding regions from the *X*. *fastidiosa* polygalacturonase might produce a soluble and biologically active chimeric protein in *E*.*coli*. Such a chimera would allow us to assess the catalytic site amino acids of *X*. *fastidiosa* polygalacturonase and confirm these amino acids are responsible for catalyzing the degradation of polygalacturonic acid substrate. Furthermore, producing a second chimera, including both the catalytic amino acids as well as the QMK substrate binding domain amino acids, would determine if, indeed, the QMK domain specific amino acids are enzymatically relevant for substrate binding. Moreover, if the *X*. *fastidiosa* polygalacturonase QMK motif was mutated to the RLK motif found in all other characterized active polygalacturonases, then a more biologically active enzyme might be observed. If this was the case these active chimeric proteins could function as surrogates for native *X*. *fastidiosa* polygalacturonase in *in vitro* enzyme inhibition assays.

## Materials and Methods

### Bacterial strains, plasmids, and PCR primers

All bacterial strains, plasmids and PCR primers used in this study are presented in Table 1.

**Table 1:** Bacterial strains, plasmids and PCR primers.

Strain, Plasmid or Primer	Characteristics and Sequences	Source
Strains		
*E*. *coli* Top10	F– *mcr*A Δ(*mrr*-*hsd*RMS-*mcr*BC) Φ80*lac*ZΔM15 Δ*lac*X74 *rec*A1 *ara*D139 Δ(*ara leu*) 7697 *gal*U *gal*K*rps*L (StrR) *end*A1 *nup*G	Invitrogen
*E*. *coli* BL21 (DE3)	F- *omp*T *hsd*S_B_ (r_B_- rn_B_-) *gal dcm* (DE3)	EMD
*Xylella fastidiosa*	Fetzer	Napa
*Agrobacterium vitis*	Strain S4	Burr
Plasmids		
pCR2.1-TOPO	*neo* (Kan^R^) *bla* (Amp^R^), *lacZ*, T7	Invitrogen
pET 30b	*neo* (Kan^R^) T7 promoter	EMD
pCR2.1XFPG	pCR2.1-TOPO vector with *pglA* gene	This study
pCR2.1AVPG	pCR2.1-TOPO vector with *pehA* gene	This study
pET30bXFPG	pET 30b with *pglA* gene in frame with T7 promoter and plasmid encoded his tag	This study
pET30bAVPG	pET 30b with *pehA* gene in frame with T7 promoter and plasmid encoded his tag	This study
pFLAG-ATS	Shuttle vector for chimera construction	Sigma
pFA-AvPG	Shuttle vector for chimera construction	This study
pBSSK	Shuttle vector for chimera construction	
pBS-AvPGbx	Plasmid used in the fusion of *pglA* gene and *pehA* gene	This study
pBS-AvPGbxX1A	pBS-AvPGbx with 840–1044bp fragment of *pglA* gene replacing 840–1044bp fragment of *pehA* gene	This study
pBS-AvPGbxX1A	pBS-AvPGbx with 840–1191bp fragment of *pglA* gene replacing 840–1191bp fragment of *pehA* gene	This study
pET30b-AX1APG	pET30bAVPG with 840–1044bp fragment of *pglA* gene replacing 840–1044bp fragment of *pehA* gene	This study
pET30b-AX2APG	pET30bAVPG with 840–1191bp fragment of *pglA* gene replacing 840–1191bp fragment of *pehA* gene	This study
pET30b-AX2APG Q380R	pET30b-AX2APG point mutation Q380R	This study
pET30b-AX2APG M381I	pET30b-AX2APG point mutation M381I	
pET30b-AX2APG Q380R M381I	pET30b-AX2APG double point mutation Q380R M381I	This study
Primers		
XFPGF	CATATGAACCTTGACCGTTT	This study
XFPGRH	CTCGAGGATAGGCGAATCAGGAAAT	This study
AVPGF	CATATGCCCGGACCTGTTTTTGC	This study
AVPGRH	GTCGACAAACGGCGACCCATCCA	This study
XPG1R_nco	GCGCCATGGCCATAGCCAAAGTGGTTGTGTAAGAAGC	This study
XPG2R_sph	GCGGCATGCATATTTTGGAGTAGGTGACATGGTC	This study
APGF-cla	CGCATCGATGGACGCGGCGGTTCGCTGCTGTTGTCC	This study
ntOLR	CCATCGGTATTTTTGACCGTATCAGGCGTGAAACAGGTGGCGGGCGTCAG	This study
ntOLF	ACGGTCAAGAACACCGATGGCTTTGATCCTGGGCAATCAAACCACGTG	This study
SXPGa1139g	CTAACAGCAATGGTTTGCGGATGAAATCTGATGCCGA	This study
ASXPGa1139g	TCGGCATCAGATTTCATCCGCAAACCATTGCTGTTAG	This study
SXPGg1143a	ACAGCAATGGTTTGCAGATAAAATCTGATGCCGATCATG	This study
ASXPGg1143a	CATGATCGGCATCAGATTTTATCTGCAAACCATTGCTGT	This study
SXPGa1139g_g1143 a	CCTAACAGCAATGGTTTGCGGATAAAATCTGATGCCGATCATG	This study
ASXPGa1139g_g1143a	CATGATCGGCATCAGATTTTATCCGCAAACCATTGCTGTTAGG	This study
T7 promoter	TAATACGACTCACTATAGGG	
T7 terminator	GCTAGTTATTGCTCAGCGG	
AVPGM	GTTCGCTGCTGTTGTCCGGC	This study

### Polygalacturonase gene cloning

*X*. *fastidiosa* was grown on solid PD3 [22] medium at room temperature for 7 days. Colony PCR amplification of the *pglA* gene (gene ID:1142827) was performed using Platinum *taq* polymerase (Invitrogen, Carlsbad, CA) and *pglA* specific primers XFPGF and XFPGRH with the following PCR parameters: 94°C (2 min) followed by 35 cycles of 94°C (1 min) 60°C (1 min) 72°C (2 min) and a final extension of 72°C (6 min). The resulting 1,635 bp PCR product was gel purified with the QIAquick gel extraction kit (Qiagen, Venlo, The Netherlands), cloned into pCR2.1 plasmid using the TOPO-TA cloning kit (Invitrogen, Carlsbad, CA) and transformed into *E*. *coli* TOP10 cells. The resulting plasmid, pCR2.1XFPG, was digested with *Nde*I and *Xho*I (NEB, Ipswich, MA) restriction enzymes and ligated with T4 DNA ligase (NEB, Ipswich, MA) into the *Nde*I and *Xho*I sites of pET30B (EMD Milipore, Billerica, MA) such that a 6X His tag sequence was present on the 3’ end of the gene, creating pET30BXFPG. *A*. *vitis* strain S4 (kindly provided by T. Burr, Cornell, NY) was grown on solid 523 medium [23] at room temperature for 48 hours. Colony PCR amplification and cloning of the *pehA* gene (GenBank: ACM36064.1) was performed exactly as stated above for *X*. *fastidiosa* except the *pehA* specific primers, AVPGF and AVPGRH, were used in the PCR reaction. The resulting 1,635 bp PCR amplicon was gel-purified as described above, cloned into pCR2.1 using the TOPO-TA cloning kit and transformed into *E*. *coli* TOP10 cells (Invitrogen, Carlsbad, CA). The resulting plasmid pCR2.1AVPG, was digested with *Nde*I and *Sal*I restriction enzymes (NEB, Ipswich, MA) and ligated with T4 DNA ligase (NEB, Ipswich, MA) into the *Nde*I and *Xho*I sites of the pET30B (EMD Milipore, Billerica, MA) resulting in the plasmid pET30BAVPG.

### *X*. *fastidiosa*/*A*. *vitis* chimera creation

The pBS-AvPGbx plasmid was generated as the backbone for the two chimeras. This plasmid was created in the following manner; the *X*. *fastidiosa pglA* gene was cloned into pBSSK via the shuttle vector pFLAG-ATS. Plasmid pET30bAVPG was digested with *Nde*I and *Xho*I restriction enzymes and the resulting full length *A*. *vitis pehA* gene was ligated into the *Nde*I and *Xho*I sites of pFLAG-ATS creating pFA-AvPG. The pFA-AvPG plasmid was then digested with *BamH*I and *Xho*I enzymes and ligated into the *BamH*I and *Xho*I sites of pBSSK, creating pBS-AvPGbx. To create the chimeric genes and ligate the products into the pET30b expression vector, an overlap extension PCR procedure was performed. Two sections of the *pglA* gene, X1 and X2, were PCR-amplified from plasmid pET30BXFPG with the following primers: the catalytic domain “X1” (877–1044 bp) was amplified with the XPG1R_Nco and ntOLF primers and the catalytic and substrate binding domain “X2” (877–1191 bp) was amplified with the XPG2R_Sph and ntOLF primers. Similarly, a portion ‘A’ of the *pehA* gene (536–880bp) from the pBS-AvPGbx plasmid was amplified with the APG1F_Cla and ntOLR primers. Both PCR reactions were performed using the following parameters: PCR was performed using Herculase II fusion DNA polymerase (Agilent, Santa Clara, CA) and the cycling parameters were 94°C (1 min) followed by 35 cycles of 94°C (15 sec) 60°C (15 sec) 72°C (1 min) and a final extension of 72°C (6 min). These two PCR reactions generate fragments with a region of 100% sequence identity between the fragments that can then be used in an overlap extension PCR which uses homologous recombination to individually fuse the X1 and X2 portions of the *pglA* gene to that of the “A” portion of the *pehA* gene piece. The overlap extension PCR using the above described primers was performed with Herculase II enzyme with the following cycling parameters: 94°C (1 min) followed by 10 cycles of 94°C (15 sec) 60°C (1 min) 72°C (1 min) and a final extension of 72°C (6 min) and then 94°C (1 min) followed by 25 cycles of 94°C (15 sec) 60°C (15 sec) 72°C (1 min) and a final extension of 72°C (6 min). The resulting fused PCR products were digested with *Cla*I and *Nco*I for the X1-A fusion and *Cla*I and *Sph*I for the X2-A fusion and ligated into the corresponding restrictions sites of pBS-AvPGbx to create the plasmids pBS-AvPGbxX1A and pBS-AvPGbxX2A. The nucleotide sequences of gene fusions were confirmed by sequencing with primer ACPGM. The full-length chimeric genes were excised from pBS-AvPGbxX1A and pBS-AvPGbxX2A plasmids as *Nde*I and *Xho*I fragments and subsequently ligated into pET30b creating the chimeric expression plasmids pET30b-AX1APG and pET30b-AX2APG, respectively.

### Point mutation creation

All mutants created in this study were generated by creating point mutations in the plasmid pET30b-AX2APG. Point mutations were created with the QuikChange Lightning Multi Site-Directed Mutagenesis Kit (Agilent, Santa Clara, CA) according to the manufacturer’s instructions. Briefly, 50 ng of pET30b-AX2APG plasmid were incubated with 125 ng of the mutation primer pair in a reaction tube containing appropriate buffers and QuikChange Lightning enzyme. This reaction mixture was used in the following PCR reaction: 95°C (2 min) followed by 10 cycles of 95°C (20 sec) 60°C (10 sec) 72°C (4 min) and a final extension of 72°C (5 min). Following amplification, the samples were incubated with the restriction enzyme *Dpn* I. During this step any parental plasmids are degraded, whereas plasmids containing point mutations are not digested because these plasmids are not *dam* methylated and, thus, resistant to *Dpn* I digestion. The resulting plasmids with the introduced mutations were then transformed into *E*. *coli* and screened by sequencing with primer AVPGM to identify plasmids that contained genes having the desired mutations. Plasmids with the proper mutations were then transformed into BL21 (DE3) for subsequent protein expression. The following primers were used in the point mutation reaction described above to create the desired mutated plasmids. To create a glutamine to arginine mutation at position 380 the SXPGa1139g and ASXPGa1139g primer pair was used as described above to generate pET30b-AX2APG Q380R. To create an isoleucine mutation at position 381, the SXPGg1143a and ASXPGg1143a primer pair was used as described above to generate pET30b-AX2APG M381I. Lastly, to create the double mutant glutamine to arginine mutation at position 380 and methionine to isoleucine mutation at position 381, the SXPGa1139g_g1143 a and ASXPGa1139g_g1143a primer pair was used as described above to generate pET30b-AX2APG Q380R M381I. The nucleotide sequences of all plasmids were confirmed by sequencing with the T7 promoter and terminator primers, as well as, the AVPGM primer.

### Protein expression and purification

Proteins containing the previously described mutations were expressed in the BL21 (DE3) *E*. *coli* strain. BL21(DE3) transformed with each plasmid was grown on an orbital shaker 250 RPM at 37°C for 16 hours in 500 ml Luria broth plus kanamycin 50 μg/ml (LB + kan50). Exponentially growing cells, OD600 = 0.8, were induced by the addition of 1mM IPTG and incubated on an orbital shaker set to 250 RPM and 37°C for 3 hours. Cells were harvested by centrifugation at 4,000 × g, suspended in 15 ml of lysis buffer (50 mM NaH_2_PO_4_, 300 mM NaCl, 10 mM imidazole pH 8.0, 0.25 mg/ml lysozyme), incubated on ice for 30 min. The cells were disrupted by sonication with a model 100 sonic dismembrator (ThermoFisher, Waltham, MA) at a power setting of 8 using six repetitions of ten second bursts. Cells were then centrifuged at 12,000 × g for 10 minutes at 4°C, and the soluble fraction was removed and incubated with 1 ml of NI-NTA agarose beads (Qiagen, Venlo, The Netherlands) for 1 hour at 4°C. The NI-NTA agarose beads were then loaded onto a chromatography column and washed with 15 ml of wash buffer (50 mM NaH_2_PO_4_, 300 mM NaCl, 50 mM imidazole pH 8.0). Proteins were eluted from the column in 500 μl aliquots of elution buffer (50 mM NaH_2_PO_4_, 300 mM NaCl, 250 mM imidazole pH 8.0). Fractions containing 58 kDa recombinant polygalacturonase, as determined by SDS polyacrylamide gel electrophoresis, were used in subsequent experiments.

### Enzymatic activity assays

Polygalacturonic acid (PGA, Sigma P-3889, Sigma-Aldrich, St. Louis, Mo) was used as substrate in all enzymatic activity assays. Polygalacturonase activity assay was monitored as follows: each 200 μl reaction contained 175 μl of 200 mM sodium acetate pH 5.0 and 25 μl of His column purified polygalacturonase enzyme adjusted where applicable to 5 U/ml (One unit will release 1.0 μmole of reducing sugar per min at pH 5.0 at 37°C). Reactions were incubated at 37°C for 1–16 hours. Polygalacturonase activity was determined by measuring the accumulation of reducing sugars using the 2-cyanoacetamide method [24]. The reaction was stopped by mixing 100ul of enzymatic reaction with 450 μl 100 mM borate buffer pH 9.0 and 100 μl of a 1% 2-cyanoacetamide solution and incubated at 95°C for 15 min. The solution was cooled to room temperature at which point the concentration of reducing sugars was estimated by measuring the absorbance of the solution at 276 nm.

## Results and Discussion

### *X*. *fastidiosa*/*A*. *vitis* protein chimeras

**Fig 1:**
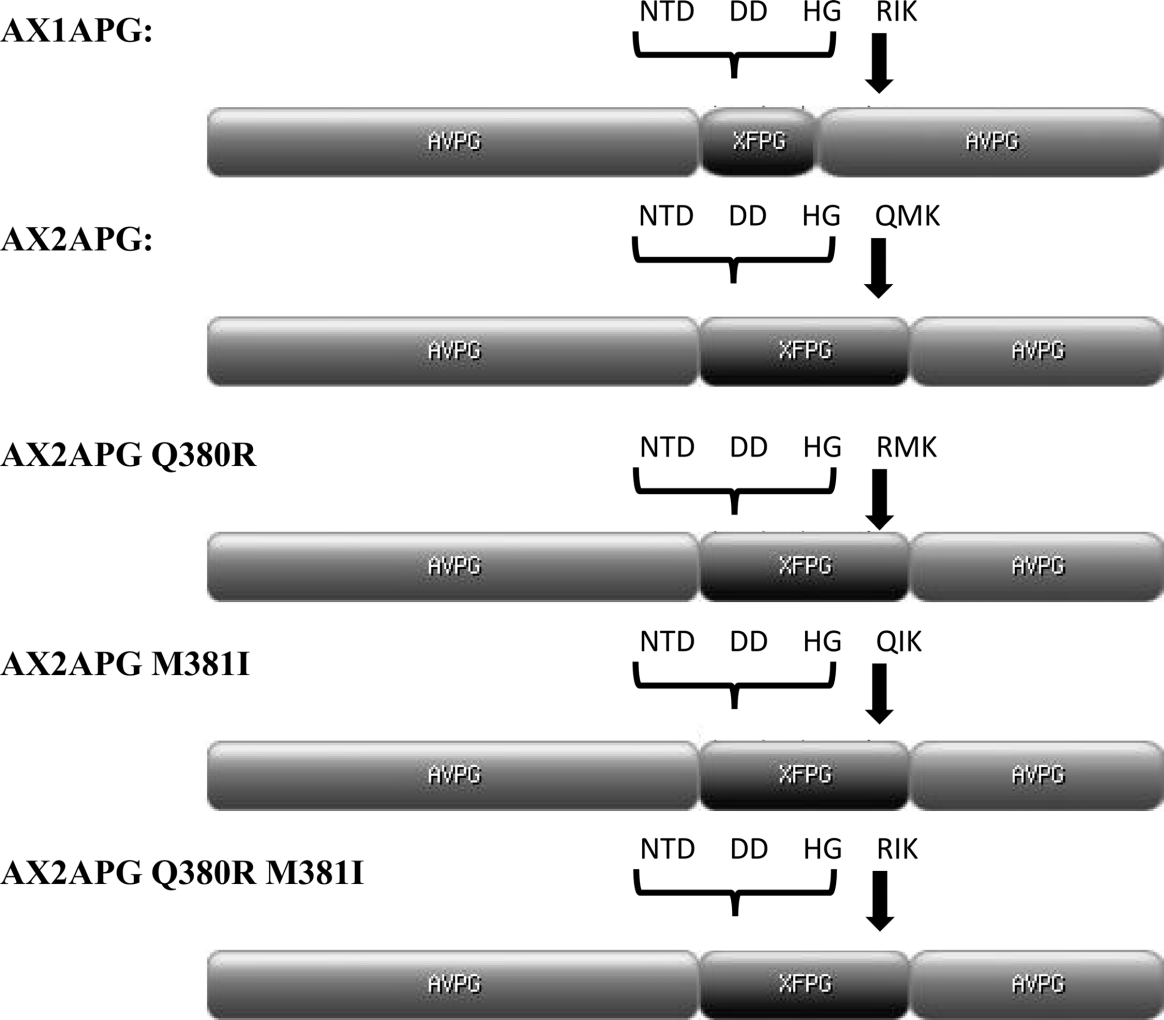
Gene diagrams for each of the chimera constructs. The AX1A chimera was constructed to specifically test if recombinant chimeric protein, produced in *E*. *coli*, is soluble and if the *X*. *fastidiosa* polygalacturonase catalytic amino acids are able to hydrolyze polygalacturonic acid. The AX2A chimera was constructed to evaluate the ability of the unique QMK motif of *X*. *fastidiosa* polygalacturonase to bind to and allow the hydrolysis of polygalacturonic acid. Notice the differing substrate binding motifs RIK in *A*. *vitis* polygalacturonase and QMK in *X*. *fastidiosa* polygalacturonase. The AX2A chimera was also used to construct the QMK to RMK, QIK, and RIK point mutations to test for substrate binding and hydrolysis. Point mutations of the QMK to RIK motif are denoted by the arrows. The NTD, DD, and HG are conserved catalytic motifs conserved in bacterial endopolygalacturonases. Diagrams made with the MyDomains program (http://prosite.expasy.org/cgi-bin/prosite/mydomains/).

Two *X*. *fastidiosa / A*. *vitis* polygalacturonase chimeras (AX1A, AX2A) were generated to investigate if the *X*. *fastidiosa* polygalacturonase active site amino acids mediated the hydrolysis of polygalacturonic acid, and to determine the biological relevance of the unique QMK substrate binding site of *X*. *fastidiosa* polygalacturonase (Fig 1).

**Fig 2:**
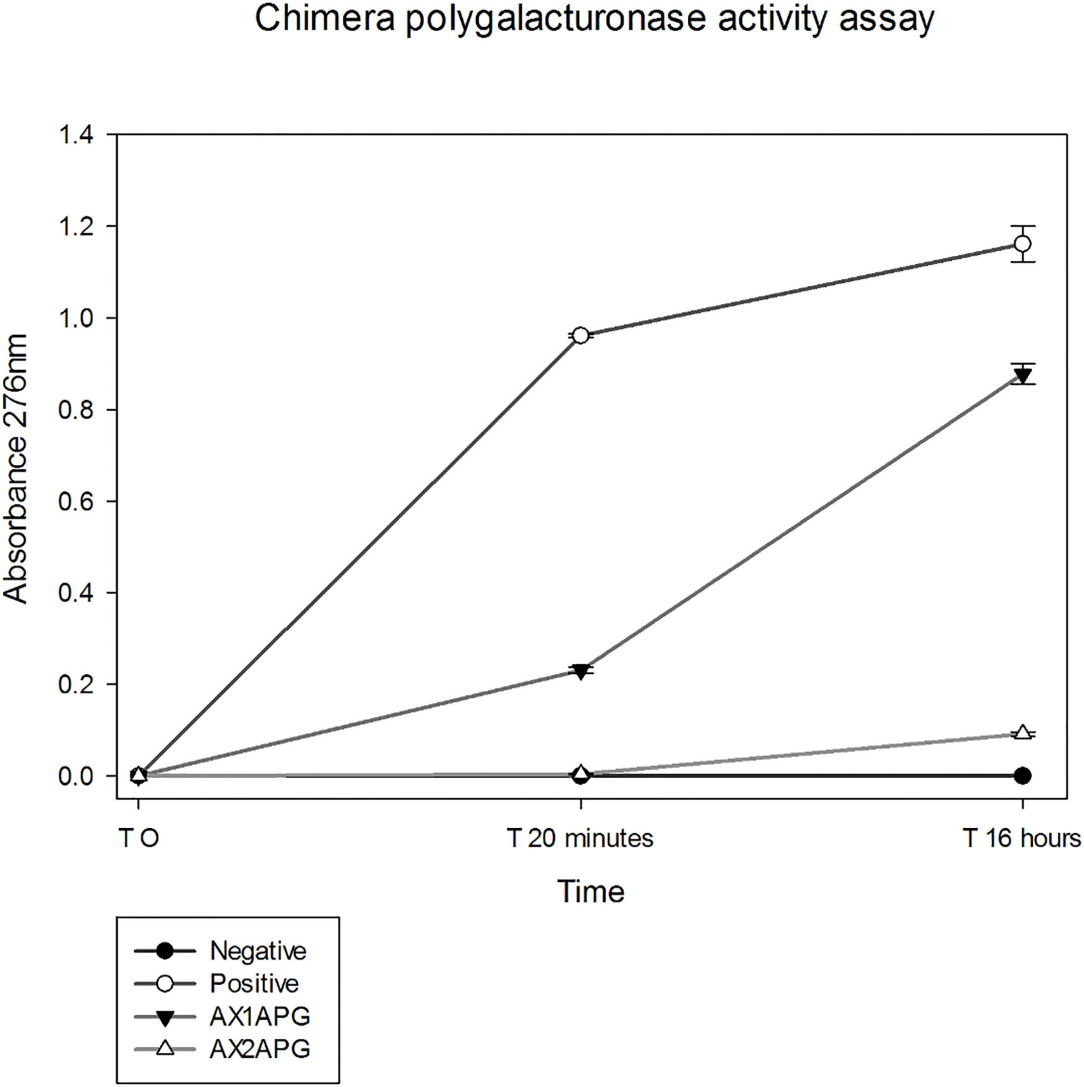
2-cyanoacetamide reducing sugar assay measuring the increase in reducing ends produced via hydrolysis of PGA substrate produced by polygalacturonase enzymatic activity. Both polygalacturonase chimeras, AX1APG and AX2APG, show the ability to hydrolyze the PGA substrate significantly greater than the negative control, albeit at a reduced level compared to the positive control *A*. *vitis* polygalacturonase. AX1APG hydrolyzed substrate greater than AX2APG. Negative control consists of enzyme mixture with no polygalacturonase added. Data was analyzed using one-way analysis of variance and multiple pairwise comparisons using the Tukey test P = 0.05 (SigmaPlot). Data are represented as the mean of three replicates and error bars represent the 95% confidence interval.

The *X*. *fastidiosa / A*. *vitis* polygalacturonase chimera AX1A contained the N-terminal and C-terminal portions of *A*. *vitis* polygalacturonase and the *X*. *fastidiosa* polygalacturonase catalytic amino acids but did not include the unique QMK substrate binding motif of *X*. *fastidiosa* polygalacturonase. The AX1A chimera was constructed to specifically test if recombinant chimeric protein, produced in *E*. *coli*, is soluble and if the *X*. *fastidiosa* polygalacturonase catalytic amino acids are able to hydrolyze polygalacturonic acid. The AX2A chimera contained the N-terminal and C-terminal portions of *A*. *vitis* polygalacturonase and the *X*. *fastidiosa* polygalacturonase catalytic amino acids as well as the unique QMK substrate binding motif of *X*. *fastidiosa* polygalacturonase and was constructed to evaluate the ability of the unique QMK motif of *X*. *fastidiosa* polygalacturonase to bind to and allow the hydrolysis of polygalacturonic acid. Chimeras AX1A and AX2A were produced in an *E*. *coli* protein expression system and both chimeric proteins were present and purified from the soluble fraction. The elutions containing soluble AX1A and AX2A chimeras were then used in 2-cyanoacetamide reducing sugar activity assays to determine solubility and activity. The results of the AX1A chimera activity assay showed that the active site amino acids of *X*. *fastidiosa* polygalacturonase hydrolyzed 1, 4-alpha-D-galactosiduronic linkages of polygalacturonic acid (Fig 2). Additionally, the results of the AX2A chimera activity assay shows that the AX2A chimera displays a markedly reduced catalytic activity compared to the AX1A chimera (Fig 2). This suggests that the QMK binding motif of *X*. *fastidiosa* polygalacturonase is less efficient in mediating enzymatic activity as previous research suggested when a R to Q mutation in *A*. *niger* polygalacturonase resulted in only 6.5% residual activity [15, 16].

**Fig 3:**
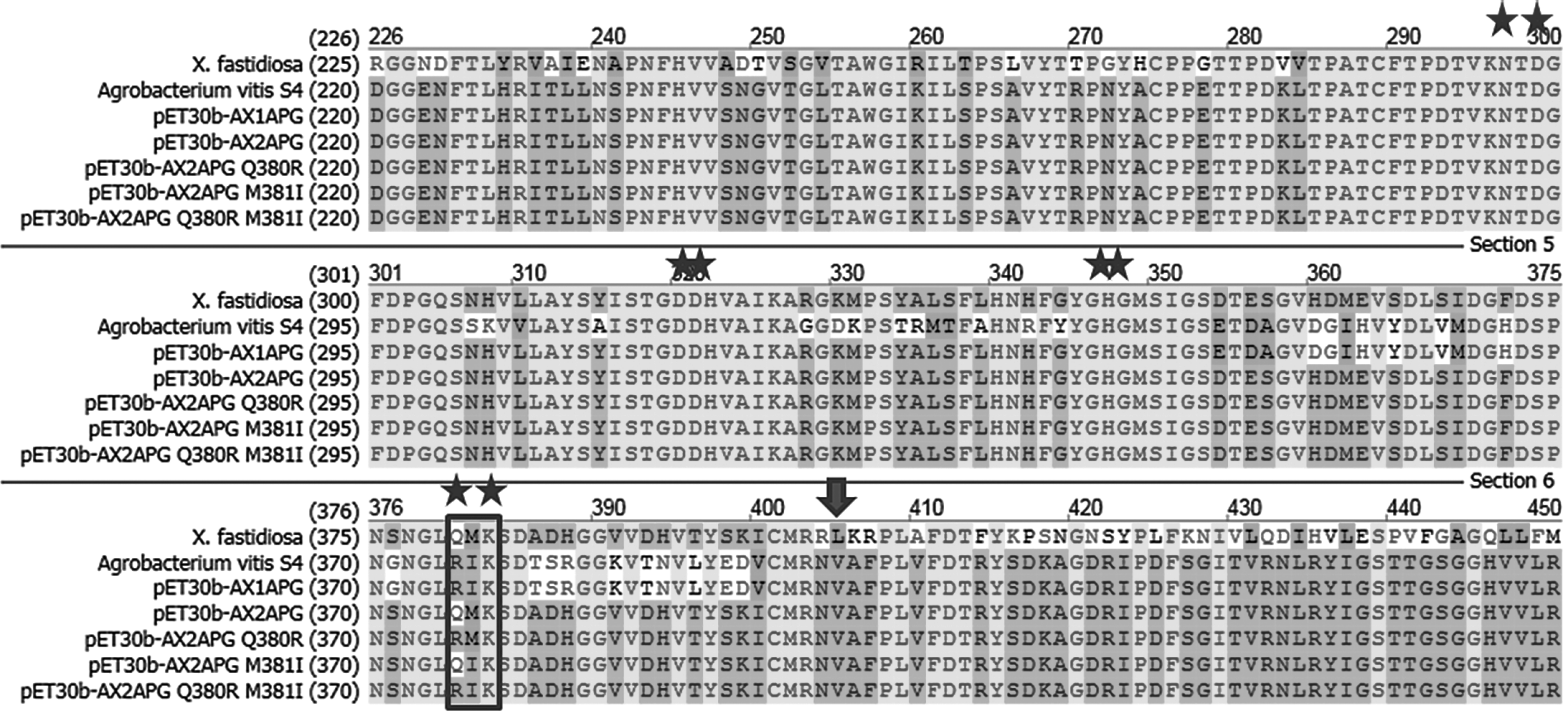
Sequence alignment of *X*. *fastidiosa* and *A*. *vitis* polygalacturonases, the AX1APG and AX2APG chimeras and the point mutants: pET30b-AX2APG Q380R, pET30b-AX2APG M381I, and pET30b-AX2APG Q380R M381I. Amino acids conserved in all polygalacturonases (N,D,D,D,F,G,H,R,K) including the R-Q mutation in *X*. *fastidiosa* polygalacturonase are denoted by stars, the locations of the point mutations are boxed and the RLK motif unique to *X*. *fastidiosa* polygalacturonase is denoted by the arrow.

To further investigate the role of the unique QMK substrate binding motif in *X*. *fastidiosa* polygalacturonase enzymatic activity, three mutants were constructed using the AX2APG chimera to determine if changing this motif to the conserved RIK motif would result in increased enzymatic activity (Fig 3).

**Fig 4:**
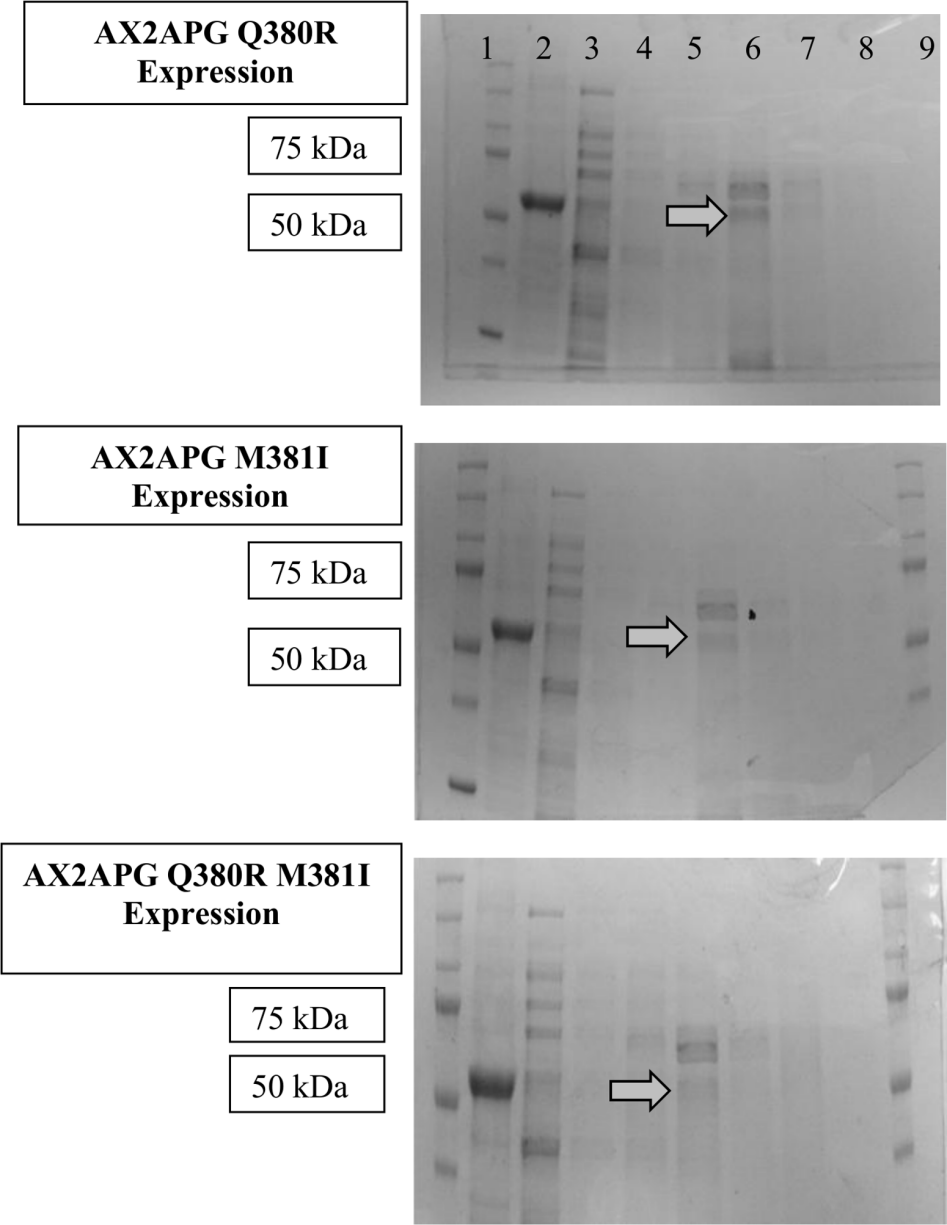
Coomassie stained SDS-PAGE gel analysis of point mutation purified chimeric proteins. All gels were loaded in the same manner. Lane 1: Dual color protein ladder (BioRad, Hercules, CA), Lane 2: His column purified *A*. *vitis* polygalacturonase positive control, Lane 3: His column purification flow-through, Lane 4: His column purification wash, Lanes 5–8: His column elutions notice lane 6 (Elution 2) containing chimeric protein bands denoted by arrows. Lane 9, if applicable, dual color protein ladder (BioRad, Hercules, CA).

**Fig 5:**
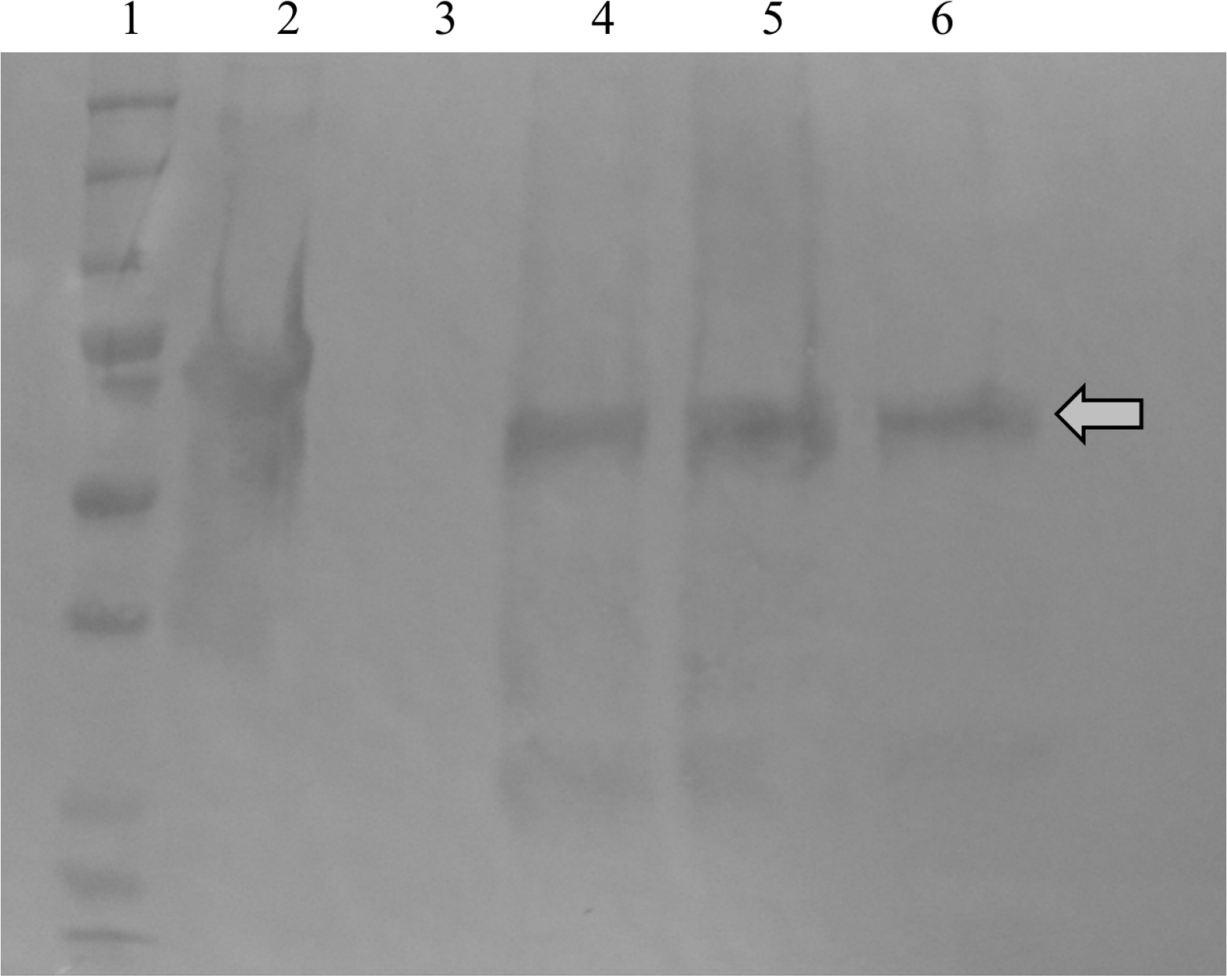
Western blot analysis of point mutation purified chimeric proteins using polyclonal anti *X*. *fastidiosa* polygalacturonase antibodies. Arrow denotes expected 58 kDa chimeric protein bands. Lane 1: Dual color protein ladder (BioRad, Hercules, CA), Lane 2: His column purified *X*. *fastidiosa* polygalacturonase positive control, Lane 3: AX2APG Q380R His column wash (negative control containing no detectable chimeric polygalacturonase), Lane 4: His column purified AX2APG Q380R elution #2, Lane 5: His column purified AX2APG M381I elution #2, Lane 6: His column purified AX2APG Q380R M381I elution #2.

The first point mutation constructed, AX2APG Q380R, changed the Q residue in the motif to a R residue, resulting in a RMK motif. The second point mutation, AX2APG M381I, changed the M residue to an I residue, resulting in a QIK motif. The third point mutation generated, AX2APG Q380R M381I, changed the Q residue to an R residue and the M residue to an I residue, resulting in the conserved RIK motif. The RIK motif was chosen, as this is the conserved substrate binding motif in *A*. *vitis* polygalacturonase. Each point mutation construct was expressed in an *E*. *coli* protein expression system, and each of the mutant proteins were isolated and purified from the soluble fraction (Figs 4 and 5).

**Fig 6:**
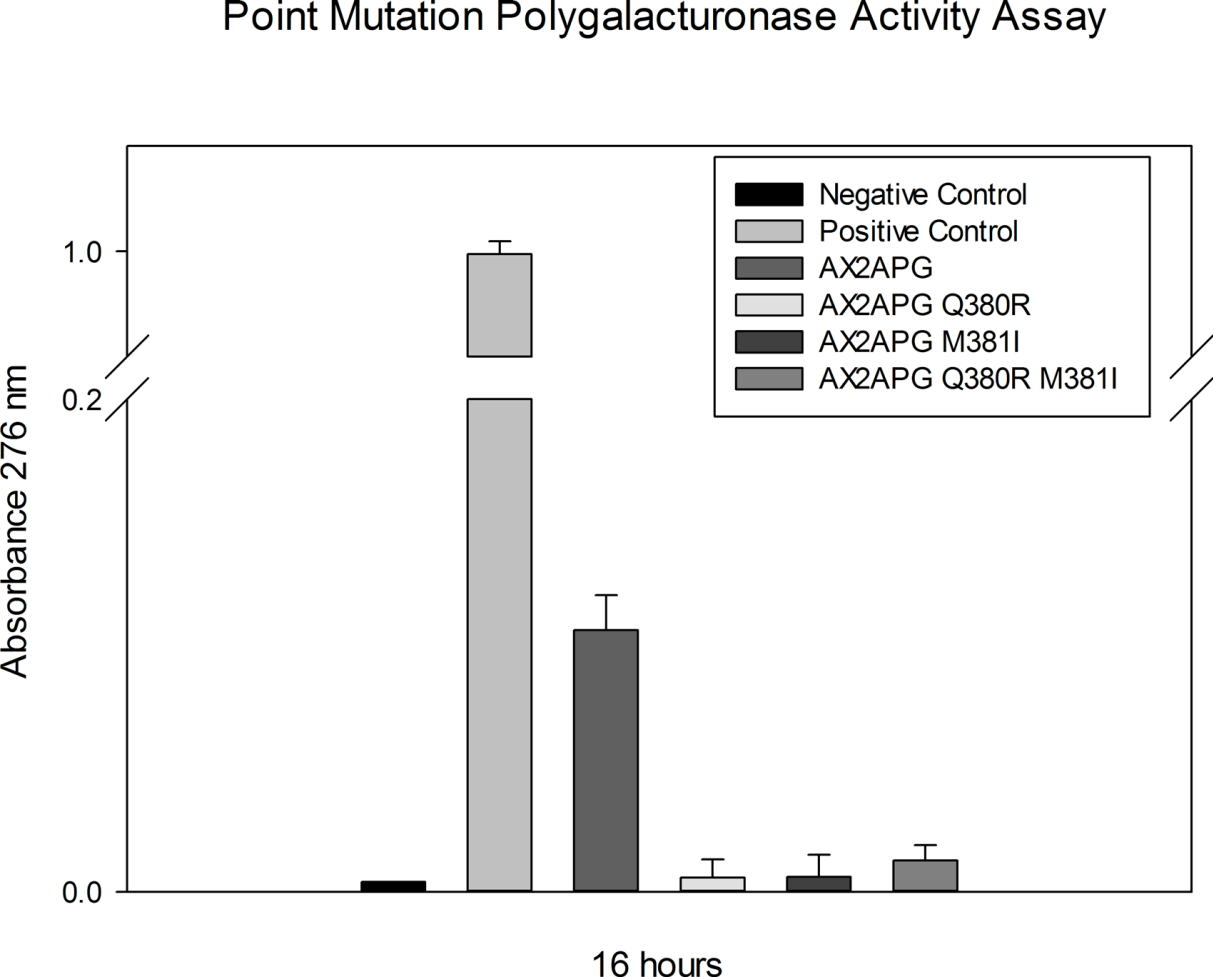
2-cyanoacetamide reducing sugar assay measuring the increase in reducing ends produced via hydrolysis of PGA substrate produced by polygalacturonase enzymatic activity. None of the polygalacturonase mutant chimeras, AX2APG Q380R, AX2APG M380I, or AX2APG Q380R M381I, shows the ability to hydrolyze the PGA substrate. Only the AX2APG chimera containing the wild-type *X*. fastidiosa QMK motif shows enzymatic activity significantly greater than the negative control. Positive control is *A*. *vitis* polygalacturonase and negative control consists of enzyme mixture with no polygalacturonase added. Data was analyzed using one-way analysis of variance and multiple pairwise comparisons using the Tukey test P = 0.05 (SigmaPlot). Data are represented as the mean of three replicates and error bars represent the 95% confidence interval.

The results of the activity assays with the mutants were surprising in that none of the mutants showed any activity in 2-cyanoacetamide reducing sugar activity assays (Fig 6). This is exactly the opposite result that would be expected from previous polygalacturonase mutational studies which suggest a mutation of an R residue to a Q residue would reduce activity to 6.5% of wild type [15, 16]. These results suggest that the QMK motif may be required for the architecture of the *X*. *fastidiosa* polygalacturonase binding cleft and subsequent enzymatic activity.

Interestingly, rhamnogalacturonase, another plant cell wall degrading enzyme responsible for degradation of “hairy pectin” where the rhamnogalacturonan has a large number of branched side chains possesses a K residue (K253 *Aspergillus aculeatus* numbering) that is thought to be involved in carbohydrate substrate binding. Specifically the K253 residue of rhamnogalacturonase is thought be involved in binding galacturonic acid as well as rhamnose in the rhamnogalacturonan chain [25, 26]. This result led us to re-evaluate the amino acids of the active cleft of *X*. *fastidiosa* polygalacturonase to determine if *X*. *fastidiosa* polygalacturonase may have other substrate binding motifs located in different positions. Indeed, it was determined that there is in fact a RLK motif near the C-terminal end of the binding site. This motif is in a position where it could possibly interact with the substrate and is not found in this position in any other polygalacturonases. While we cannot rule out the possibility that this chimera is constraining the substrate binding cleft in some manner, leading to reduced activity, the fact that AX2A is active and none of the mutants show activity supports our conclusion that the QMK motif is biologically important for the activity of *X*. *fastidiosa* polygalacturonase. These results also suggest that *X*. *fastidiosa* polygalacturonase could have a preference for a different pectic substrate or, at least, that it has a different manner of substrate binding than other polygalacturonase enzymes characterized to date.

In conclusion, these experiments showed that it was possible to generate *X*. *fastidiosa / A*. *vitis* polygalacturonase chimeras that are active and soluble proteins in *E*. *coli* expression systems. Additionally, the catalytic amino acids of *X*. *fastidiosa* polygalacturonase are able to hydrolyze 1, 4-alpha-D-galactosiduronic linkages of polygalacturonic acid. Some evidence is also presented that the *X*. *fastidiosa* polygalacturonase may possess unique substrate binding amino acids. These chimeras should be able to be used in phage panning experiments to select for inhibitors that target the active site of the enzymes.

## Supporting Information

S1 Text Data sets for Fig 2 and Fig 6. (XLSX)
